# The Early Diagnosis of Uterine Cancer1Read before the Physician’s League, October 31, 1898.

**Published:** 1899-03

**Authors:** Maude J. Frye

**Affiliations:** Buffalo, N. Y.


### THE EARLY DIAGNOSIS OF UTERINE CANCER.[1]

BASED ON TWENTY-FOUR CASES SEEN IN THE SERVICE OF DR. FREDERICK, AT THE BUFFALO WOMAN'S HOSPITAL.

By MAUDE J. FRYE, M. D., Buffalo, N. Y.

THE cases on which my paper is based are those which have come under my personal observation since my connection with the Buffalo Woman's Hospital, into which institution they had come for treatment. They include the cases which have been treated in the hospital during this time, a period of about twenty months, but not others which may have come for diagnosis, nor with one exception, those occurring in Dr. Frederick's practice outside the hospital. Before taking up the question of early diagnosis there are other points of interest connected with these cases which I wish to present.

The average age of the patient was approximately 51½ years. Seven were under 45, three under 40, the youngest being 34. The oldest patient was 68. Nineteen of the patients had borne children. Of the five nulliparous women it is interesting to note that cancer began in the cervix in three cases. One of these, however, gave a history of a miscarriage, at what month not stated.

The previous health of women developing cancer is usually good, contrasting forcibly with other gynecological cases. Five cases gave a history of cancer in the family, two of the mother, two of the sisters, one of the father. In eleven cases the cancer attacked the cervix, in ten of them being so far advanced when seen that the diagnosis was made macroscopically. Total hysterectomy was done in one case of cervical cancer, symptoms of which had been present five months, and the diagnosis of which was made by microscope. The growth returned and death of the patient followed within ten months of operation—an illustration of the extreme malignancy of some cases. A second case illustrates this even better. Seventeen months after an operation for double ovarian cyst, a patient presented herself for examination, symptoms of trouble having been present for a month. On examination an inoperable cancer, originating apparently in the cervix, but infiltrating the body, was found.

The majority of cases of cancer of the cervix were too far advanced for anything but palliative treatment. Amputation was

1. Read before the Physician's League, October 31, 1898.

done four times, one of these cases being reported well after fifteen months. Eleven cases were of cancer of the endometrium. In nine of these cases total hysterectomy was done. One patient died one week after operation of septic peritonitis, caused by infection of the peritoneum through the accidental opening of the uterine cavity during operation. A second patient has died of a return of the cancer, a third, though still alive, is suffering from a return. Three are well after periods ranging from ten to fourteen months. The others are so recent that it is too early to speak of results. In one case of endometrial cancer the operation was begun, but the patient not bearing the anesthetic well, it was discontinued after the tying of one uterine and both ovarian arteries. This patient is well after eighteen months. In one case the cancer had returned in the vaginal scar, a total hysterectomy having been done at another hospital six months before. I have included in this list a case of multiple sarcoma, and one of lymphadenoma, which properly do not belong here. The former case was a woman of 42 years, mother of two children; previous health, fair; family history good. She had been ailing for six years, and during the last four years she had suffered from uterine hemorrhage. Previous to curetting no positive diagnosis was made. She requested the doctor while she was under the anesthetic to remove from the scalp two wens of considerable size. Curetting showed a suspicious growth in the endometrium. While under the anesthetic a lump of uncertain nature, attached to the sacrum, was made out. On cutting down upon one of the supposed wens, a malignant growth, leading down to and through the cranium, was discovered. The microscope completed the diagnosis of sarcoma.

The case of lymphadenoma has been included in those of cancer of the endometrium. It is one of those borderline cases shading into malignancy. Curetting having failed to relieve, total hysterectomy was done. The patient is now well, over a year after operation.

It is almost impossible to overstate the insidious nature of uterine cancer. The most constant early symptom, a bloody discharge from the vagina, is sometimes so slight as scarcely to attract the attention of the patient. If the cancer develops before the menopause any disturbance of menstruation is at once accounted for by the patient or her friends, in that universal explanation, "It's the change of life." The phenomena attributed by the female lay members of the community to the menopause would require a large volume for their description. On no subject of physiology is ignorance fraught with such deplorable consequences. Teach every woman that any increase in the monthly flow at this time, or at any time, is abnormal, as is a longer duration than usual, nor should there be any show of blood between the periods. A woman at the menopause may normally skip one period or more and at the next menstruation flow to make up for the missed periods; anything more than this is pathological. It is not entirely, indeed it is hardly ever, the fault of the physician that cancer of the cervix is seen too late for operation.

In nineteen of the twenty-four cases here reported a vaginal discharge, usually stated as being distinctly bloody, was the first symptom noted. In one case this bloody discharge began during the woman's fifth pregnancy, at which time the malignant nature of the cause of the hemorrhage might carelessly be attributed to something else not so unusual. The patient came to the hospital nine months after the birth of her child, only to be told that temporary relief was all that could be given. One patient reported that the first symptom observed by her was an occasional blood-stain upon her clothing. She consulted her physician, who disregarded the symptoms until nearly or quite a year later. This scanty and occasional discharge of blood, with a moderate loss of flesh, were the only symptoms observed in her case during the thirteen months of the disease. Looking over the case histories, I find that this symptom varies from a scanty and occasional show to an almost constant bloody discharge. The flow is seldom excessive early in the history, as is the flow in fibroma. In one case loss of flesh had been noticed for one year, the discharge of blood for three months only. This patient was distinctly cachectic. In the second case the patient had been ailing for one year, but the uterine discharge had existed for one month only. Pain is the unusual early symptom. In one case of cancer of the cervix, the patient stated it to be the first thing noticed. Often in cancer of the endometrium it has not been noticed at all. Always it is late. When pain is observed in cancer of the uterus the cases are usually past help. Pain in cancer of the endometrium does not occur as a rule until the disease has spread beyond the uterus.

*Diagnosis.*—Cancer of the cervix, as usually seen, is easy of diagnosis. Cases are not often seen in which the help of the microscope is needed. The reason for this is that these cases usually occur before the menopause and the patient disregards her symptoms till late.

The following description of early cervical cancer is taken from Penrose :

When cancer begins in an erosion of a laceration, we find that the eroded surface bleeds more easily than in the nonmalignant condition, and is somewhat more elevated than the surrounding surface of the cervix. We may by palpation detect around the erosion a more or less indurated edge, which is not felt around a benign erosion. The submucous structures of the cervix may feel brawny and indurated. If the erosion has become an ulcer, the indurated edges and the involvement of the deeper structures of the cervix are more marked. It must always be remembered that an ulcer of the cervix is very rare as a benign condition.

In the vegetating form of cancer of the cervix we may find small warty growths, or large cauliflower masses, or rounded or irregular protuberances, growing from the surface of the cervix. There is here also felt an induration around the base of the growth and throughout the cervix.

A very striking characteristic of cancerous growths of the cervix is their friability. The warty growths or cauliflower-like masses break off readily upon even gentle palpation and profuse bleeding often results.

When the disease begins immediately within the external os, this opening becomes enlarged, the cervical canal is destroyed, and there is presented the appearance of a deep conical excavation with ulcerated, unhealthy edges in the center of the vaginal cervix. When the disease begins still higher up, the cervical canal may be the seat of extensive destruction of tissue before any lesion is visible below the external os.

When the disease begins in the racemose glands of the cervix, the nodules may be felt beneath the mucous membrane of the vaginal aspect of the cervix. The whole cervix is usually indurated and somewhat enlarged. The mucous membrane overlying the nodule may appear congested, and upon palpation it is found that the underlying mucous membrane does not glide readily over the nodule, but seems to be more than normally adherent to the underlying structures.

In all forms of cancer of the cervix there is present to a greater or less extent a general induration of the cervix. The elasticity or resiliency of the cervix is diminished or lost; this is shown not only by the sensation upon palpation, but by the fact that the cervix is not capable of dilatation, by tent or otherwise, as in the normal condition.

The diagnosis in a doubtful case may readily be established by excising a small piece of tissue and examining by microscope, a practice which is always followed at the Woman's Hospital.

Endometrial cancer oftenest develops in women past the menopause. When a woman past the menopause presents herself with a history of an occasional scanty flow of blood, or possibly a constant bloody flow, often with no other symptoms, she is to be examined most carefully. Sometimes, happily, the cause of the trouble will be found external to the uterus, as for instance an abrasion of the mucous membrane at the entrance to the vagina. But should careful search discover no such cause, explain to the patient or her family the possible serious nature of the case, and insist on an examination under an anesthetic with the curette, to secure scrapings for microscopical examination. The ordinary vaginal examination in these cases of endometrial cancer reveals nothing in the early part of the disease. The cervix is normal to sight and touch, the uterus not enlarged. In curetting a case of cancerous nature one obtains much more débris than in a case of ordinary endometritis, and to the initiated the diagnosis can almost be made without the microscope. Scrapings for examination should be preserved in a 1 per cent. solution of formalin.

Never be deceived by the apparent good health of these women into thinking that cancer is out of the question. They are often, indeed perhaps usually, women with every appearance of health, a fact which misleads them as well as their physicians. Many of these cases are treated for months and even for years before the true nature of the disease is suspected, and this by good physicians.

224 ALLEN STREET.

